# Accuracy of neck circumference in the diagnosis of overweight in children

**DOI:** 10.1590/1806-9282.20240049

**Published:** 2024-07-19

**Authors:** Guilherme de Azevedo Traebert, André Legat Albino, Milcia Almeida Zaidan, Franciane Bobinski, Francieli Pereira Ribeiro, Eliane Traebert, Jefferson Traebert

**Affiliations:** 1 Universidade do Sul de Santa Catarina, School of Medicine – Palhoça (SC), Brazil; 2 Universidade do Sul de Santa Catarina, Post-Graduation Program in Health Sciences – Palhoça (SC), Brazil.

**Keywords:** Pediatric obesity, Body mass index, Diagnosis

## Abstract

**OBJECTIVE:**

The objective of this study was to estimate the accuracy of measuring neck circumference as a diagnostic method for overweight in 10-year-old children.

**METHODS:**

A cross-sectional diagnostic accuracy study was performed in 2019. The population was composed of 942 school children from the municipality of Palhoça, SC, Brazil. For each measurement of the neck circumference, sensitivity, specificity, positive and negative predictive values, likelihood ratio for a positive test, and accuracy were estimated using the receiver operator characteristic curve, with body mass index as a reference.

**RESULTS:**

The estimated overall accuracy was 88.9%. For males, the accuracy was 90.1%, and for females, 88.5%. A 30.0 cm neck circumference had a sensitivity of 22.8%, a specificity of 95.4%, a positive predictive value of 76.6%, a negative predictive value of 65.3%, a likelihood ratio for a positive test of 5.0, and an accuracy of 66.7% for all students.

**CONCLUSION:**

Neck circumference showed a global accuracy of 88.9% as a method for diagnosing overweight in 10-year-old children. Predictive values showed high values, mainly starting with a neck circumference of 30 cm.

## INTRODUCTION

Childhood obesity is a global problem. The prevalence of obesity in Brazil has been increasing gradually, with epidemic behavior in both adults and children^1^. However, a recent systematic review^2^ has shown data on childhood obesity in Brazil that cannot be generalized due to the large methodological differences between the studies.

Several parameters are used to classify overweight and obesity, but the body mass index (BMI) is more commonly used in adults, even though it does not differentiate adipose tissue from lean mass, thus not being fully correlated with body fat^1,3^. This index is calculated by the ratio between the individual's weight and height squared (kg/m^2^)^3^.

In children and adolescents aged 5–19 years, overweight and obesity are characterized by BMI percentile curves or z-scores. The World Health Organization^3^ defines overweight as a BMI situated on z-score curves between values 1 and 2 for age. The obesity classification corresponds to the BMI located on the curve above the value 2. Such values differ with age according to the variation in corpulence, which is understood as different dimensions that the body assumes at different ages during growth^1,3^.

On the contrary, other diagnostic methods have been proposed to help measure childhood obesity, such as waist circumference^4^ and neck circumference^5^. The latter showed an important correlation with metabolic syndrome parameters and has a very low cost^6^.

Studies^4,5,7^ have shown a relationship between childhood overweight and obesity and neck circumference, being potentially as effective as waist circumference and BMI in measuring overweight and obesity. In 6-year-old children, neck circumference presented an accuracy of 77.2% as a diagnostic method for overweight. A positive linear correlation of 0.57 was observed between neck circumference and BMI. Sensitivity and specificity values were low, but high positive predictive values were observed, particularly in 30- and 31-cm neck circumference measurements^6^.

Although there is some evidence about the accuracy of neck circumference as a diagnostic measure of obesity in 6-year-old children, no references were found about such indicators beyond that age. Therefore, the objective of this study was to estimate the accuracy of the neck circumference measurement as a method for diagnosing overweight in 10-year-old children.

## METHODS

This is a cross-sectional diagnostic accuracy study. The information was obtained from the database of the *Coorte Brasil Sul* study^8^ involving 10-year-old school children from 37 public and 19 private schools in Palhoça/SC, Brazil. The study population consisted of data from 942 children. The parameters used to calculate the sample size were as follows: population of 1,270 10-year-old children, expected prevalence of unknown outcome (p=50%), 95% confidence level, and 2% relative error, which generated a minimum sample of 831 children. That number was beefed up by 10% to compensate for refusals, which generated a final sample of 942 children randomly selected from all schools.

Data collection was carried out directly in the schools by two surveyors who participated in the training and calibration process for anthropometric data collection. The training of the surveyors was carried out based on joint training, observing the variation in anthropometric data obtained simultaneously by both surveyors. In the second step, calibration was carried out by collecting data from 30 children of 10 years old to check inter-examiner and intra-examiner reproducibility. The agreement of weight, height, and neck circumference measurements was evaluated using the kappa test. All values were greater than 0.7, which was considered an adequate agreement.

Weight and height were collected following the norms proposed by the Brazilian Ministry of Health^9^. The anthropometric assessment was performed using BMI obtained by dividing the weight in kilograms by height in meters squared^10^. The cutoff points in the BMI z-score were as follows: normal weight (≥ −2 and <+1), overweight (≥ +1 and <+2), and obesity (≥ +2)^10^. Neck circumference was measured in centimeters using a measuring tape. The child remained upright with the head positioned in the horizontal plane. The upper edge of the measurement tape was positioned just below the cricothyroid cartilage and surrounded perpendicularly the neck.

Specific data for this investigation (gender, BMI, and neck circumference measurements) were entered into an Excel spreadsheet and subsequently exported to the Statistical Package for Social Sciences (SPSS) 18.0 software, which was used for data analysis.

For each measurement of the neck circumference, sensitivity, specificity, and positive and negative predictive values were estimated, as well as the likelihood ratio for a positive test and accuracy. BMI was used as a reference. Accuracy was calculated by the ratio between the sum of true positives and true negatives in the total sample. Accuracy was expressed by the receiver operator characteristic (ROC) curve and its relevant confidence interval. Additionally, the correlation between neck circumference and BMI was reviewed using Pearson's correlation test. All measures were estimated for the population as a whole and by gender. Measures that had p-values <0.05 were considered statistically significant.

The study was approved by the Ethics Committee for Research in Humans of the Universidade do Sul de Santa Catarina under number 3.362.267.

## RESULTS

The prevalence of excess weight was 39.6% (95%CI 36.5–42.5). Neck circumference ranged from 21 to 38 cm, with a mean of 28.8 cm (SD=2.2), a median of 28.0 cm, and a mode of 29.0 cm.

**Figure 1:**
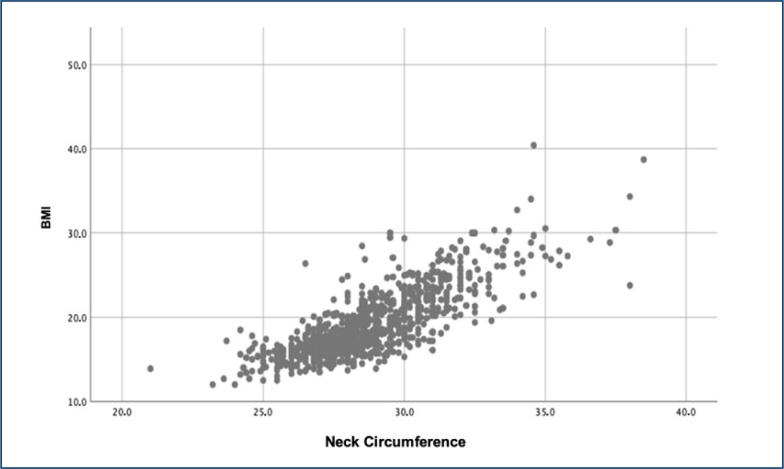
Correlation between measures of neck circumference (cm) and body mass index. Palhoça (SC), Brazil (n=942).

The relationship between neck circumference and BMI is shown in Figure 1. A positive and statistically significant correlation was observed (p<0.001; Pearson's correlation coefficient, r=0.751; and determination coefficient, R^2^=0.564).

The neck circumference values, as well as the sensitivity, specificity, positive and negative predictive values, the likelihood ratio for the positive test, and data accuracy according to sex are presented in Table 1. A neck circumference of 29.0 cm had a sensitivity of 19.3%, a specificity of 87.5%, and an accuracy of 60.5% for all students. The positive and negative predictive values were 50.3 and 62.3%, respectively. The likelihood ratio for the positive test showed that it is 1.6 times more likely to find a 29 cm neck circumference among overweight children when compared with children without excess weight (Table 1).

**Table 1:** Sensitivity, specificity, predictive values, likelihood ratio for the positive test, and accuracy of neck circumference measurements as a diagnostic method for overweight by gender in 10-year-old school children, Palhoça (SC), Brazil (n=942).

Accuracy measures for the male						Accuracy measures for the female						
NC (cm)	S (%)	Sp (%)	PPV (%)	NPV (%)	LR+	A (%)	S (%)	Sp (%)	PPV (%)	NPV (%)	LR+	A (%)
23.0	–	99.2	–	58.3	–	58.0	–	99.3	–	62.0	–	61.2
24.0	–	99.6	–	58.4	–	58.2	–	94.8	–	60.9	–	58.9
25.0	–	98.1	–	58.0	–	57.3	–	89.5	–	59.6	–	55.7
26.0	–	84.8	2.4	54.5	–	49.8	2.2	80.4	6.3	57.5	0.1	50.8
27.0	2.1	74.5	5.6	51.7	–	44.4	6.5	68.0	10.9	54.5	0.2	44.7
28.0	10.7	67.7	19.0	57.6	0.3	44.0	17.2	81.7	36.4	61.9	0.9	57.3
29.0	15.0	83.7	39.4	58.0	0.9	55.1	23.7	90.8	61.1	66.2	2.6	65.4
30.0	24.1	94.3	75.0	63.6	4.2	65.1	21.5	96.4	78.4	66.9	6.0	68.1
31.0	18.2	98.1	87.2	62.8	9.6	64.9	13.4	99.0	89.3	65.3	13.7	66.7
32.0	13.9	100.0	100.0	62.0	–	64.2	8.1	100.0	100.0	64.2	–	65.2
33.0	7.0	100.0	100.0	60.2	–	61.3	2.7	100.0	100.0	62.8	–	63.2
34.0	8.6	100.0	100.0	59.4	–	60.9	3.2	100.0	100.0	63.0	–	63.4
35.0	2.1	100.0	100.0	59.0	–	59.3	1.1	100.0	100.0	62.4	–	62.6
36.0	0.5	100.0	100.0	58.6	–	58.7		–	–	–	–	–
37.0	0.5	100.0	100.0	58.6	–	58.7	0.5	100.0	100.0	62.3	–	62.4
38.0	1.6	100.0	100.0	58.8	–	59.1	–	–	–	–	–	–

NC: neck circumference; S: sensitivity; Sp: specificity; PPV: positive predictive value; NPV: negative predictive value; LR+: likelihood ratio for the positive test; A: accuracy.

The neck circumference value of 30.0 cm had a sensitivity of 22.8%, a specificity of 95.4%, a positive predictive value of 76.6%, a negative predictive value of 65.3%, a likelihood ratio for the positive test of 5.0, and an accuracy of 66.7% for the entire group of school children. The sensitivity and specificity values for males were 24.1 and 94.3%, and the accuracy was 65.1%. The positive and negative predictive values were 75.0 and 63.6%, respectively. The likelihood ratio for the positive test showed that it is 4.2 times more likely to find an overweight male child with a neck circumference of 30 cm when compared with those without excess weight (Table 1). On the contrary, in females, the sensitivity and specificity values were 21.5 and 96.4%, and the accuracy was 68.1%. The likelihood ratio for the positive test showed that it is 6.0 times more likely to find an overweight female child with a neck circumference of 30 cm when compared with those without excess weight (Table 1).

**Figure 2:**
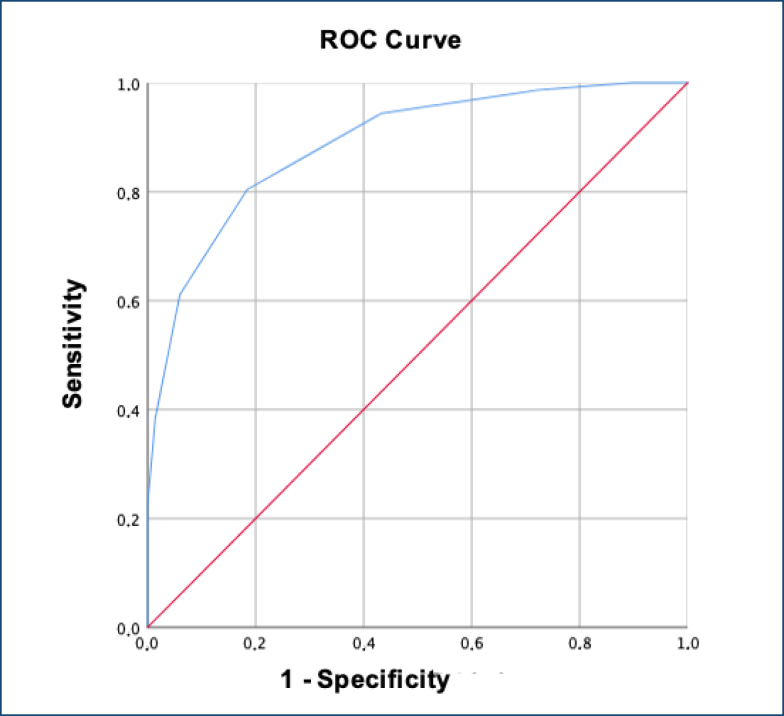
Receiver operator characteristic curve of neck circumference measurements (cm) as a diagnostic method for overweight in 10-year-old school children. Palhoça (SC), Brazil (n=942).

The ROC curve is shown in Figure 1. The area under the curve corresponds to the accuracy of neck circumference as a method for diagnosing overweight. The accuracy value of the global ROC curve was 88.9% (95%CI 86.7–90.9), p<0.001 (Figure 2). The accuracy for males was 90.1% (95%CI 87.2–92.9), p<0.001 (Figure 2), and it was 88.5% (95%CI 85.6–91.5), p<0.001 in females.

## DISCUSSION

As a representative measure of fat deposition in the upper body, neck circumference is a new, pathogenic, and independent fat deposit, which is related to the rate of visceral fat and may be associated with greater cardiovascular risks compared with fat in the central region of the body^11^. This is because subcutaneous fat in the upper body region supplies most of the free fatty acids to the systemic circulation in post-absorptive and postprandial conditions and can cause disorders such as hypertriglyceridemia^12^.

A Brazilian study^5^ carried out with 6-year-old children demonstrated an accuracy of 77.2% as a diagnostic measure to identify overweight and obesity, while this study showed a greater accuracy of 88.9%, albeit with 10-year-old children. Regarding sensitivity, both studies showed low values; at 6 years of age, sensitivity reached a maximum of 23% in children with a neck circumference of 27 cm. On the contrary, this study detected a maximum sensitivity value of 24.1% when the neck circumference value was 30 cm in males. Regarding specificity, both studies showed high values. When comparing this variable in children with a neck circumference of 31 cm, our study presented 98.6% sensitivity, while the study with 6-year-old children presented 99.0% sensitivity. In the aforementioned study, the positive predictive values in young girls with a neck circumference of 30 and 31 cm presented values of 77 and 85% sensitivity, respectively. On the contrary, for males, the values were 85 and 93% for boys having 30 and 31 cm neck circumference. Despite evaluating students of a different age, this study presented positive predictive values of 75.0 and 87.2% with 30 and 31 cm in males, respectively. In females, the values were 78.4% with 30 cm and 89.3% with a neck circumference of 31 cm.

A similar Chinese study^13^ evaluated 3,719 children aged 7–10 years. It reported a significant positive correlation between neck circumference and obesity at all ages and in both genders. Accuracy was 70% and the optimal neck circumference to diagnose overweight/obesity in boys was between 24.75 and 27.25 cm, while for girls, it was between 23.75 and 26.25 cm. According to the authors, the best measurement to estimate excess weight was 29 cm neck circumference, which had a sensitivity of 19.3%, a specificity of 87.5%, a positive predictive value of 50.3%, a negative predictive value of 62.3%, and an accuracy of 60.5% for all children.

Another study^14^ in the United States involving 1,102 children aged 6–18 years showed a positive correlation in both boys and girls, with a higher number of older children. In that study, a neck circumference of 28.5–39 cm indicated high BMI in boys and 27–34.6 cm high BMI in girls. In 10-year-old boys, overweight could be diagnosed from 32 cm neck circumference onward with 94% accuracy, 85.7% sensitivity, and 95.2% specificity. On the contrary, in 10-year-old girls, overweight could be diagnosed starting with 30.5 cm with 79.9% accuracy 79.9% sensitivity, and 70.3% specificity. Pearson's correlation index between BMI and neck circumference in 10-year-old children was 0.71 and 0.78 for boys and girls, respectively. In comparison, our study presented a 0.75 Pearson global correlation index. Also, in the Michigan study^14^, globally, the measure to estimate excess weight was 29 cm, with a sensitivity of 15.0%, a specificity of 83.7%, a positive predictive value of 39.4%, a negative predictive value of 58.0%, and an accuracy of 55.1% in boys and 23.7, 90.8, 61.1, 66.2, and 65.4% in girls, respectively.

A meta-analysis study^15^ with children and adolescents between 6 and 18 years demonstrated moderate accuracy for diagnosing overweight and obesity in this population because the accuracy value estimated by the global ROC curve was 87.0%, which corroborates with the result of this study, in which the global accuracy value was 88.9%.

The predictive values found in this study were higher than the sensitivity and specificity values. For clinical practice, predictive values are more useful^16^ as they indicate the probability of the event assessed to occur; specifically, in this case, overweight at 10 years of age, considering the results of the diagnostic test.

Thus, the proportion of female children with a positive test result who were overweight was 78.4 and 89.3% with neck circumference measurements of 30 and 31 cm, respectively. In males, the proportion was 75 and 87% with neck circumference measurements of 30 and 31 cm, respectively. Thus, it can be observed that the higher the BMI, the greater the predictive values found, which is in line with another study that confirms that neck circumference is a reliable measure for the diagnosis of overweight and obesity in children^17^. It is important to emphasize that for the same test, the greater the prevalence of the event, the greater the positive predictive value and the lower the negative predictive value, which is extremely important in the case of childhood obesity, as this is a highly prevalent event^16^. In addition, the values of the likelihood ratios for the positive test at 30 cm of neck circumference showed a good probability of finding overweight children, which were 4.2 times in males and 6.0 times in females.

A possible limitation of this study is the fact that more than one surveyor collected the data, which could, eventually, produce measurement bias. However, training exercises made it possible to measure the inter-examiner and intra-examiner reproducibility. Furthermore, the standardization and strict adherence to the collection methods ensure the reliability of the results.

It can be concluded that the neck circumference showed a global accuracy of 88.9% as a method for diagnosing overweight. Predictive values showed high values, mainly starting with a neck circumference of 30 cm.

## References

[1] Associação Brasileira para o Estudo da Obesidade e da Síndrome Metabólica (2016). Diretrizes Brasileiras de Obesidade.

[2] Heinz C, Afonso L, Traebert E, Trevisol DJ, Traebert J (2022). Prevalência de excesso de peso corporal infantil no Brasil: uma revisão sistemática. Res Soc Develop.

[3] World Health Organization (2024). Obesity and overweight.

[4] Traebert E, Leão G, Traebert GA, Flôres APR, Traebert J (2022). Accuracy of abdominal circumference for diagnosing overweight in six-to-seven-years-old children. Res Soc Develop.

[5] Mucelin E, Traebert J, Zaidan MA, Piovezan AP, Nunes RD, Traebert E (2021). Accuracy of neck circumference for diagnosing overweight in six- and seven-year-old children. J Pediatr (Rio J).

[6] Mendes CG, Barbalho SM, Tofano RJ, Lopes G, Quesada KR, Detregiachi CRP (2021). Is neck circumference as reliable as waist circumference for determining metabolic syndrome?. Metab Syndr Relat Disord.

[7] Kelishadi R, Djalalinia S, Motlagh ME, Rahimi A, Bahreynian M, Arefirad T (2016). Association of neck circumference with general and abdominal obesity in children and adolescents: the weight disorders survey of the CASPIAN-IV study. BMJ Open.

[8] Traebert J, Lunardelli SE, Martins LGT, Santos KD, Nunes RD, Lunardelli AN (2018). Methodological description and preliminary results of a cohort study on the influence of the first 1,000 days of life on the children's future health. An Acad Bras Cienc.

[9] Ministério da Saúde do Brasil (2024). Secretaria de Atenção à Saúde.

[10] World Health Organization (2024). Body mass index (BMI).

[11] Preis SR, Massaro JM, Hoffmann U, D'Agostino RB, Levy D, Robins SJ (2010). Neck circumference as a novel measure of cardiometabolic risk: the Framingham Heart study. J Clin Endocrinol Metab.

[12] Jensen MD (2008). Role of body fat distribution and the metabolic complications of obesity. J Clin Endocrinol Metab.

[13] Hu NN, He M, Li YF, Wang RY, Yang J, Li WJ (2022). Correlation of neck circumference with body mass index in preschool children. Zhongguo Dang Dai Er Ke Za Zhi.

[14] Nafiu OO, Burke C, Lee J, Voepel-Lewis T, Malviya S, Tremper KK (2010). Neck circumference as a screening measure for identifying children with high body mass index. Pediatrics.

[15] Ma C, Wang R, Liu Y, Lu Q, Liu X, Yin F (2017). Diagnostic performance of neck circumference to identify overweight and obesity as defined by body mass index in children and adolescents: systematic review and meta-analysis. Ann Hum Biol.

[16] Medronho R, Bloch KV, Luiz RR, Werneck GL (2009). Epidemiologia.

[17] Taheri M, Kajbaf TZ, Taheri MR, Aminzadeh M (2016). Neck circumference as a useful marker for screening overweight and obesity in children and adolescents. Oman Med J.

